# Physicians’ gender and specialty in relation to adverse drug reaction reporting in Sweden

**DOI:** 10.1007/s00228-026-04077-9

**Published:** 2026-05-08

**Authors:** Lina-Maria Nordvall, Bo Rolander, Maria Larsson, Linnea Melin, Staffan Hägg, Anders Kling

**Affiliations:** 1 Department of Medical and Translational Biology, Clinical Pharmacology and Pharmacology, Umeå University, Umeå, Sweden; 2 Futurum-Academy for Healthcare, Jönköping, Sweden; 3 Medical Products Agency, Uppsala, Sweden; 4 Department of Medical and Health Sciences, Linköping University, Linköping, Sweden; 5 Department of Clinical Science, Child and Adolescent Psychiatry, Umeå University, Umeå, Sweden

**Keywords:** Pharmacovigilance, Adverse drug reaction reporting systems, Sex factors, Physician-patient relations, Specialties, medical, Physicians, Time factors, Gender concordance

## Abstract

**Purpose:**

To evaluate the possible influence of gender and specialty on adverse drug reaction (ADR) reporting among physicians before and after the COVID-19 pandemic.

**Methods:**

This retrospective nationwide register study analysed all ADR reports submitted by physicians to the Swedish Medical Products Agency during 2017 and 2023 (*n* = 4079 and 3740, respectively). The reporting rates were calculated and stratified by gender and specialty.

**Results:**

The highest reporting rate among physicians was observed in medical specialties, followed by primary care and psychiatry (27, 10, and 12, reports/100 physicians in 2017; 18, 15, and 9 in 2023). The lowest reporting rates were observed for surgical and hospital service specialties (8 and 1 in 2017; 6 and 1 in 2023). Male and female physicians reported ADRs to a similar extent and both reported more frequently on female patients. Gender concordance between physician and patient was associated with significantly higher reporting (*p *< 0.001 in 2017; *p* = 0.041 in 2023).

**Conclusion:**

Reporting varied across specialties, and gender concordance emerged as a previously unrecognized factor influencing ADR reporting. These findings provide new opportunities for targeted interventions to enhance physician participation in pharmacovigilance.

**Supplementary Information:**

The online version contains supplementary material available at 10.1007/s00228-026-04077-9.

## Introduction

After approval of a new drug, spontaneous ADR reporting from healthcare is one of the major tools for detecting unknown adverse effects of drugs. Despite common assumptions, knowledge about long-term safety and rare adverse effects remains limited at the time of approval.

In Sweden, as in many countries, healthcare providers are legally required to report all suspected ADRs to the Medical Products Agency [1]. However, only a fraction of the ADRs are reported [2]. Given the important role of physicians’ ADR reporting in signal detection and assessments of drug safety issues, it is desirable to increase ADR reporting among physicians. If reporting were steadily higher and more aligned with the real incidence of ADRs, potential safety issues could be detected and managed earlier. This would save lives, money and unnecessary suffering. Knowledge of factors influencing ADR reporting is essential to address underreporting.

Previous literature on factors influencing ADR reporting among healthcare professionals (HCPs) has identified knowledge gaps on what, when, and to whom ADRs should be reported as major reasons for underreporting [3]. Some misconceptions identified include only serious or unknown ADRs should be reported, only well-tolerated drugs are approved for the market, or that causality between drug and adverse reaction should be nearly established prior to reporting [3–7]. Additionally, lack of time has emerged as a major barrier to reporting [3, 6, 8]. Although less characterized, personal factors of the HCP, such as gender and specialty, have also been suggested to influence reporting propensity [9–11]. However, most previous studies are survey-based, and studies on the impact of physician gender or specialty are scarce [9, 10].

This study therefore aimed to examine possible effects of the physician’s gender and specialty on the reporting of ADRs, as well as the influence of the physician’s gender in relation to the patient’s gender on ADR reporting.

## Methods

### Data collection and analysis

All ADR reports submitted by physicians to the Swedish Medical Products Agency (MPA) in 2017 and 2023 were included in the analysis. To examine potential associations between ADR reporting propensity and specialty or gender, data from ADR reports were analysed with respect to the reporter’s workplace and gender, as well as patient gender. The original reports contain information on the ADR, suspected and concomitant drugs, temporal relationships, patient sex and age, in addition to reporter name, profession, workplace, postal address and contact details. For the purpose of this study, only information regarding physician gender, workplace and patient biological sex were extracted. Information about patients’ biological sex was available in the reports and used as the best available proxy for patient gender. Physician gender was inferred from the reporter’s first name by personnel at the MPA using national name statistics from Statistics Sweden. Only names with clear gender classification (≥ 10 individuals of the same gender in the national database) were assigned.

The number of licenced physicians in healthcare as well as the number of dispensed drugs for each of the years was obtained from Statistics from Swedish National Board of Health and Welfare. The number of ADR reports and physicians were then used to calculate reporting rates for respective year (number of ADR reports per 100 physicians), to enable comparison between genders and specialties, respectively. Statistics about licensed physicians employed in healthcare could only be stratified by gender and not by specialty or working place, as these data were unavailable. Therefore, numbers of licensed specialists in each specialty were used when comparing different specialties.

Reports in which the specialty of the physician’s workplace could not be determined were excluded from analysis concerning ADR reporting rates across specialties (*n* = 139 in 2017; *n* = 142 in 2023). Similarly, reports where the gender of the physician was unclear were excluded from the analysis concerning gender and ADR reporting rate (*n* = 41 in 2017; *n* = 29 in 2023). In addition, reports with unknown patient gender (*n* = 2 in 2017; *n* = 1 in 2023) were excluded from analysis concerning gender concordance. The number of excluded reports due to unknown patient or physician gender was 43 in 2017 and 30 in 2023.

As changes might have occurred between the pre- to post COVID-19 pandemic period in overall reporting rates and across specialties, analysis was conducted for two different full years, 2017 (three years before the pandemic) and 2023 (three years after the start of the pandemic). This also enabled specialty-stratified analyses of gender and gender concordance effects at two distinct time points. For gender concordance analysis, the percentage of all female physicians reports that concerned female patients was compared to the percentage of all male physicians reports that concerned female patients. Because comparisons were made within each specialty, overall patient gender distribution across specialties does not explain the observed differences.

The type of specialty was categorized, as previously described by Bexelius et al. with smaller modifications [12], in primary care (general practice), medical specialties (internal medicine, paediatrics, nephrology, pulmonary medicine, allergology, endocrinology, gastroenterology, geriatrics, gynaecological oncology, haematology, dermatology, infection medicine, cardiology, neurology, oncology, palliative medicine, rehabilitation medicine, rheumatology, and pain medicine), surgical specialties (emergency medicine, anaesthesiology, pediatric surgery, hand surgery, otolaryngology, general surgery, vascular surgery, neurosurgery, gynaecology and obstetrics, orthopaedics, plastic surgery, thoracic surgery, urology, and ophthalmology), psychiatry (child and adolescent psychiatry, geriatric psychiatry, and psychiatry) and hospital service specialties (laboratory medicine specialties, radiology, clinical pharmacology, occupational- and environmental medicine, and school healthcare medicine).

### Statistical analyses

All statistical analyses were conducted using SPSS version 25 (IBM Corp., Armonk, NY, USA; released 2017). The variables are presented in numbers (n) and percentages (%). To evaluate potential influence of physician–patient gender concordance on ADR reporting, the Pearson chi-square test was applied. To assess trends in ADR reporting over time, reporting rates among physicians from 2014 to 2024 were analysed with linear regression. Statistical significance was set at *p* = 0.05 (two sided).

### Ethics

The study was approved by the Swedish Ethical Review Authority (Dnr 2024-08700-01).

## Results

The total number of ADR reports submitted by physicians was 4079 in 2017 and 3740 in 2023. When adjusted for the number of licensed physicians employed in healthcare, this corresponds to 10 reports per 100 licensed physicians in 2017 and 8 per 100 in 2023 (Table 1). To assess whether the decline in ADR reporting rate from 2017 to 2023 was temporary, an eleven-year trend analysis excluding vaccine reports was performed. This analysis showed a significant downward trend in reporting rates (R2 = 0.77, *p* = 0.0004) which could not be fully explained by an increased number of licensed physicians, as the increase in number of dispensed drugs was relatively higher (S: Table 1; S: Fig. 1).

**Table 1:** Reporting rates among licensed physicians employed in healthcare

Gender of the reporting physician	Number of ADR reports 2017	Number of licensed physicians 2017	Reporting rate (ADR reports/ 100 licensed physicians) 2017	Number of ADR reports 2023	Number of licensed physicians 2023	Reporting rate (ADR reports/ 100 licensed physicians) 2023
Female	2014	19,382	10.4	1915	23,259	8.2
Male	2024	20,619	9.8	1796	22,248	8.1
Total	4038^a^	40,001	10.1	3711^a^	45,507	8.2

^a^ Reports with known gender of the physician

### Specialty and ADR reporting

When physicians’ ADR reporting was compared between specialties, the highest reporting rates were observed in rheumatology, emergency medicine, neurology, internal medicine, infection medicine, otolaryngology, dermatology, and haematology in both 2017 and 2023 (Table 2). The lowest reporting rates were observed in radiology, laboratory specialties, anaesthesiology, and several surgical specialties, which had consistently low reporting in both 2017 and 2023 (S: Table 2).

**Table 2:** Specialties with the highest reporting of ADRs when adjusted for the number of specialists in each specialty during 2017 and 2023

Specialty	Number of ADR reports 2017	% of all ADR reports 2017	Reporting rate (ADR reports/ 100 specialists) 2017	Number of ADR reports 2023	% of all ADR reports 2023	Reporting rate (ADR reports/ 100 specialists) 2023
Rheumatology	300	7.6	108.7	120	3.3	39.9
Emergency medicine	169	4.3	102.4	82	2.3	17.4
Neurology	212	5.4	45.3	189	5.3	34.2
Internal medicine	907	23.0	42.5	723	20.1	31.7
Infection medicine	184	4.7	37.8	131	3.6	23.3
Otolaryngology	200	5.1	29.2	217	6.0	28.9
Dermatology	104	2.6	24.8	121	3.4	24.3
Haematology	56	1.4	19.6	55	1.5	17.6

For full list including all specialties see S: Table 2

When categorizing specialties into larger groups, the highest reporting was observed in medical specialties in both 2017 and 2023. For all groups except GPs, a decline in reporting rates was observed from 2017 to 2023 (Table 3).

**Table 3:** Reporting of ADRs among different specialties when categorized into larger groups

Specialty categories	Number of ADR reports 2017	% of all ADR reports 2017	Reporting rate (ADR reports/ 100 specialists) 2017	Number of ADR reports 2023	% of all ADR reports 2023	Reporting rate (ADR reports/ 100 specialists) 2023
Total^a^	3940	100.0	13.5	3598	100.0	11.1
Medicine	2240	56.9	26.6	1741	48.4	18.3
Psychiatry	294	7.5	12.0	228	6.3	8.7
GPs	628	15.9	9.8	1038	28.8	14.9
Surgery	745	18.9	8.4	557	15.5	5.6
Service	33	0.8	1.1	34	0.9	1.0

^a^ ADR reports where the working place of the reporting physician could be assessed

For full list including all specialties see S: Table 2.

### Gender and ADR reporting

Male and female physicians reported ADRs to a similar extent in 2017 and 2023, and both groups reported less in 2023 compared to 2017 (Table 1). More reports were generally written for female patients. Of the ADR reports written by physicians in 2017, 57% (2321/4077) concerned female patients (*p* < 0.001). In 2023, 56% (2101/3739) of the reports were on female patients (*p* < 0.001). The gender of the patient was unknown in two ADR reports from 2017 and in one report from 2023, these were excluded from the analysis.

### Gender concordance and ADR reporting

Female physicians more frequently reported ADRs involving female patients, indicating significant gender concordance effects (Table 4). Of the ADRs reported by female physicians in 2017, 60% were on female patients, while 54% of the male physicians’ reports were on female patients. The influence of patient gender in relation to physician gender on ADR reporting was statistically significant with chi-square (*p* < 0.001). In 2023, 58% of the female physicians’ reports were on female patients, whereas 55% of the male physicians’ reports concerned female patients, still statistically significant (*p* < 0.05).

**Table 4:** Influence of reporter-patient gender relation on ADR reporting 2017 and 2023

Specialty categories	Physician gender	Patient gender and number of ADR reports 2017		Chi-square	Patient gender and number of ADR reports 2023		Chi-square
		Female (%)	Male (%)		Female (%)	Male (%)	
Total^a^	Female	1211 (60)	802 (40)	< 0.001	1107 (58)	807 (42)	0.04
	Male	1084 (54)	939 (46)		979 (55)	817 (46)	
Medicine	Female	628 (57)	479 (43)	0.4	490 (55)	399 (45)	0.5
	Male	611 (55)	501 (45)		450 (53)	393 (47)	
Surgery	Female	247 (64)	136 (36)	< 0.001	173 (55)	143 (45)	0.003
	Male	177 (51)	173 (49)		99 (42)	136 (58)	
GPs	Female	204 (68)	98 (32)	0.002	292 (64)	168 (37)	0.3
	Male	177 (55)	144 (45)		341 (60)	225 (40)	
Psychiatry	Female	90 (60)	59 (40)	0.06	96 (61)	61 (39)	0.7
	Male	71 (50)	72 (50)		40 (58)	29 (42)	
Service	Female	11 (55)	9 (45)	0.4	10 (59)	7 (41)	0.7
	Male	9 (69)	4 (31)		11 (65)	6 (35)	

Gender concordance was considered statistically significant if *p* <0.05. ^a^ADR reports with known gender of physician and patient. Reports from unknown specialities is included at total

When comparing groups of specialties, differences in ADR reporting related to physician–patient gender concordance were statistically significant for surgical specialties in both years, and for GPs in 2017. The overall trend of physicians tending to report more ADRs for patients of their own gender was consistent for both years and all groups of specialties, except for hospital service specialties (Table 4).

## Discussion

In this nationwide register study physicians’ specialty and gender in relation to patient gender were associated with differences in ADR reporting. Previous research on personal factors influencing ADR reporting is limited, largely based on surveys focusing on attitudes and barriers rather than reporter characteristics. By using national register data, this study avoids participant and recall bias and overcomes the limited power of survey designs. Unlike most prior studies, it focuses exclusively on physicians, as reasons for underreporting may vary across professional groups.

Reporting frequency varied by specialty but is likely multifactorial and should not be attributed to any single explanatory factor. Drug-intensive specialties such as rheumatology, neurology, internal medicine, and haematology showed the highest reporting rates. These differences could be related to several factors, including frequent use of high-risk drugs such as NSAIDs [13], DMARDs, biologics [14], antiepileptics [15], and anticoagulants [16]. Additionally, patients treated with these drugs often require regular contact with health care which may support detection of ADRs. High reporting rates in infection medicine and otolaryngology could be related to antibiotic-related reactions and ototoxic ADRs [17, 18], while emergency medicine and dermatology frequently encounter acute and skin-related ADRs, possibly explaining their high reporting. Immunosuppressants and biologics are increasingly used in dermatology, and ADRs are part of differential diagnostics of rashes [19]. However, these interpretations should be made with caution, as multiple interacting factors are likely involved.

Anaesthesiology, despite being drug-intensive, showed a low reporting rate (S: Table 2). This finding may reflect a combination of factors, such as the frequent use of multiple drugs and challenges in attributing causality [4, 5]. Additionally, reporting may occur in systems outside of pharmacovigilance [20, 21]. Although patient safety culture in anaesthesiology is strong, emphasis on incidents/adverse events in general, including perioperative technical problems and medication errors, may shift focus away from ADR reporting. Since perioperative ADRs are associated with high mortality, interventions that support ADR reporting in anaesthesiology are urgently needed [22, 23]. Geriatrics also had low reporting, despite high ADR risk, which may be influenced by multiple factors, including clinical complexity and assumptions that only serious or unexpected ADRs should be reported [4, 5]. Since elderly and frail patients are often underrepresented in clinical trials [24], pharmacovigilance in this population is especially critical.

Our findings regarding specialty and ADR reporting are mostly in line with previous research. In a Dutch survey, physicians were categorized as medical specialist, GPs, or surgeons. GPs were most likely to report having submitted an ADR, followed by the medical specialists [10]. In a survey from Uganda, hospital-based HCPs were grouped into medicine, paediatrics/obstetrics-gynaecology, surgery, or others. The HCPs in medicine were more likely to report at least one ADR in the past year compared to other groups [11]. Our result shows higher ADR reporting from medical specialties compared to primary care, which differs slightly from the Dutch study, whereas the Ugandan study did not compare medical specialties with primary care. Our results further show higher reporting from medical specialties compared to surgical specialties, which aligns both with the Dutch and Ugandan data.

Evidence on reporter gender effects is scarce; earlier surveys found mixed results [9, 10]. Consistent with Dutch findings, our data revealed no reporter gender differences in ADR reporting rates among physicians. Although not the scope of our study, the overall magnitude of ADR reporting on female patients was significantly higher compared to male patients, which aligns with previous research [25].

Notably, this study is the first to report an association between gender concordance and ADR reporting. While causality cannot be inferred, this finding aligns with broader literature linking gender concordance to improved clinical outcomes, including lower mortality, fewer postoperative complications, and reduced hospital readmissions e.g. [26–28]. Although not conclusive, benefits appear more pronounced for female patients [27, 29]. Women treated by male physicians have been reported to experience lower survival after acute myocardial infarction [30], receive less guideline-directed therapy for heart failure, achieve poorer glycaemic control in diabetes [27], and face greater risks of pain or stroke-risk underestimation [31–33].

Despite growing evidence, largely from North America, findings are limited by their observational designs, and not all studies align. Some European studies report no impact of gender concordance on re-hospitalization [34] or PCI outcomes [35], suggesting that differences in healthcare systems, such as universal insurance coverage, may influence results. In Sweden, patients are not generally asked about gender preferences, and we found no evidence that gender-concordant dyads are more common. In contrast, an American study showed that most patients preferred, and subsequently saw, a same-gender GP when asked beforehand [36], with stronger preferences in sexual health contexts [37].

In pharmacovigilance, women’s typically more communicative style and health-seeking behaviour may facilitate ADR reporting [38], but this advantage may diminish in gender-discordant dyads if communication barriers hinder understanding. Concordance effects likely reflect multiple mechanisms, including patient preferences, communication patterns, and physician gender biases [27, 30, 36]. Shared lived experiences may support communication in concordant dyads, whereas poor outcomes in discordant encounters may reflect insufficient gender diversity within clinical teams rather than the dyad alone [39]. Although overall concordance effects in our study were modest, they were pronounced in certain subgroups, including surgery. Increasing awareness of these patterns may help promote ADR reporting regardless of patient gender.

Our pre- to post-pandemic setting cautiously suggests relative stability in reporting patterns across gender and specialities, as the eight highest-reporting specialties were the same in both years. It also enabled speciality-stratified analyses of gender- and gender concordance effects at two different timeframes, which strengthen any interpretation of findings. However, most specialties with the notable exception of GPs, showed reduced reporting in the post- compared to pre-pandemic period. An eleven-year trend analysis revealed a significant decline in reporting rates, driven by an increase in the number of physicians rather than fewer ADR reports. Given the rise in drug use, especially among the elderly [40–44], and the growing number of drug recalls indicating persistent safety risks [45], this finding is notable and suggests increased underreporting. Although reporting has shifted from HCPs to consumers since the 2012 EU reform [46], physician reports remain crucial, and the decline is unlikely explained by increased reporting from other HCPs [47].

Some limitations of this study deserve attention. Although the sample of ADR reports were extensive, it is uncertain to what extent the selected years are representative of other time periods. Further, comparisons between specialties were dependent on the number of licensed specialists in each specialty as the number of junior physicians in each specialty was unavailable. ADR reports were, however, included independently of whether the reporter was a specialist or a junior physician working in a specific specialty. Thus, comparisons between specialties are more meaningful when grouped into broader categories. Importantly, our results regarding gender concordance do not reflect uneven patient gender distribution across specialties. The interpretation is, however, susceptible to confounding due to uneven patient gender distribution between female and male physicians. The observed effect therefore reflects both the prevalence of gender-concordant dyads *and* potential behavioural differences among physicians and/or patients in concordant versus discordant dyads. To better understand how gender, gender concordance, and specialty influence ADR reporting, further studies from diverse cultural contexts and professional groups are warranted.

## Conclusion

In this study from Sweden, physicians’ specialty and gender concordance with patients were associated with the magnitude of ADR reporting. Reporting was highest among medical specialties and higher if physicians had the same gender as their patients. Improved awareness of gender‑related and specialty‑specific patterns may enhance physician engagement in pharmacovigilance.

## Supplementary information

Below is the link to the electronic supplementary material.

Supplementary Material 1

## Data Availability

The reports investigated in this study are from the Swedish Medical Products Agency’s database for suspected adverse drug reactions, which is not publicly available as it contains personal data.
