# Supplementary material for: Physicians’ gender and specialty in relation to adverse drug reaction reporting in Sweden
Source: Eur J Clin Pharmacol. 2026 May 8;82(6):146. doi: 10.1007/s00228-026-04077-9 (PMC13152945; doi:10.1007/s00228-026-04077-9)
Supplement: Supplementary file 1 — Supplementary Material 1 [file 228_2026_4077_MOESM1_ESM.pdf]

Physicians' gender and specialty in relation to adverse drug reaction reporting in Sweden

Lina-Maria Nordvall[1], Bo Rolander[2], Maria Larsson[3], Linnea Melin[3], Staffan Hägg[2,4], Anders Kling[1,5]

[1]Department of Medical and Translational Biology, Clinical Pharmacology and Pharmacology, Umeå University, Umeå, Sweden. [2]Futurum-Academy for Healthcare, Region Jönköping County, Sweden. [3]Medical Products Agency, Uppsala, Sweden. [4]Department of Medical and Health Sciences, Linköping University, Linköping, Sweden. [5]Department of Clinical Science, Child and Adolescent Psychiatry, Umeå University, Umeå, Sweden.

Contact information: lina-maria.nordvall@umu.se

S: Table 1 Adverse drug reaction (ADR) reporting by physicians during 2014 - 2024 relative to the number of dispensed drugs and number of licensed physicians employed in health care.

Year	Number of licensed physicians	Number of Millions of dispensed drugs	Number of ADR reports (vaccine reports excluded)	Reporting rate (Number of ADR reports per 100 licensed physicians)
2014	37 362	102.9	4146	11.1
2015	38 282	105.8	4770	12.5
2016	39 164	109.1	4629	11.8
2017	40 001	112.1	3817	9.5
2018	40 935	115.2	3882	9.5
2019	40 910	119.8	3895	9.5
2020	41 533	122.7	3498	8.4
2021	42 653	125.0	3561	8.3
2022	44 367	130.3	3200	7.2
2023	45 507	135.1	3476	7.6
2024	46 048	140.9	3944	8.6

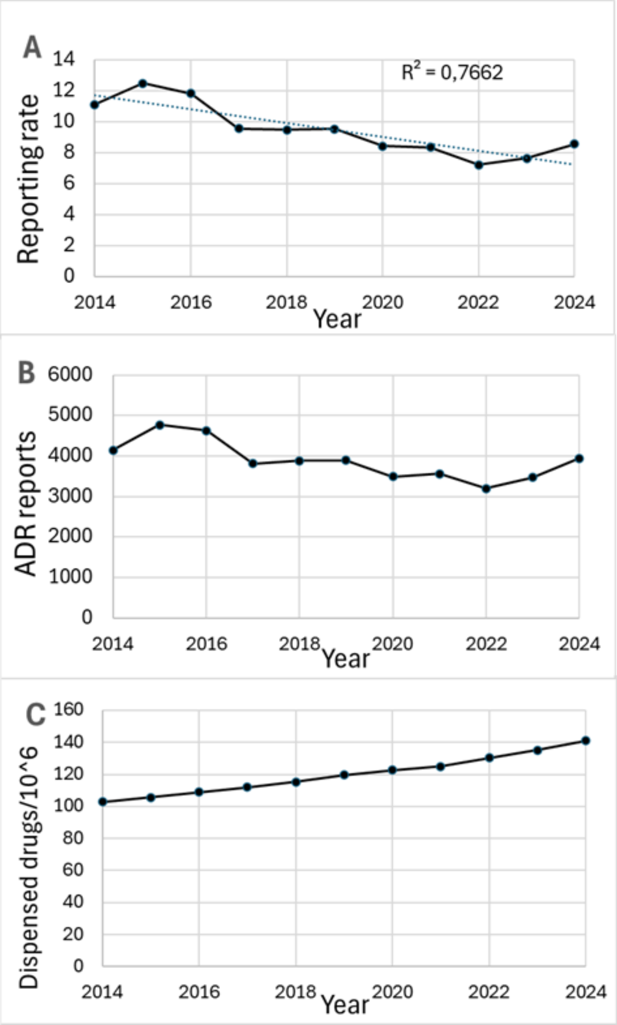

S: Fig. 1 Adverse drug reaction (ADR) reporting and number of dispensed drugs during 2014-2024.

(**A**) Reporting rates (number of ADRs reported per 100 licensed physicians).

(**B**) Absolut numbers of ADR reports submitted by physicians to the Swedish Medical Products Agency.

(**C**) Number of millions of dispensed drugs.

S: Table 2A Adverse drug reaction reporting rates (number of ADR reports per 100 specialists) among different specialties year 2017

**Specialty**	**Number of specialists 2017**	**Number of ADR reports 2017**	**% of all ADR reports 2017**	**Reporting rate 2017 (Number of ADR reports per 100 specialists)**
Rheumatology	276	300	7.6	108.7
Emergency medicine	165	169	4.3	102.4
Neurology	468	212	5.4	45.3
Internal medicine[a]	2133	907	23.0	42.5
Infection medicine	487	184	4.7	37.8
Otolaryngology[b]	685	200	5.1	29.2
Dermatology	420	104	2.6	24.8
Haematology	286	56	1.4	19.6
Oncology	479	92	2.3	19.2
Pulmonary medicine	303	56	1.4	18.5
All Specialities	29094	3940	100.0	13.5
Child and adolescent psychiatry	402	54	1.4	13.4
Psychiatry	2040	240	6.1	11.8
Paediatrics	1667	187	4.7	11.2
GPs	6404	628	15.9	9.8
Ophthalmology	773	71	1.8	9.2
Rehabilitation medicine	159	14	0.4	8.8
Cardiology	938	79	2.0	8.4
Geriatrics	537	42	1.1	7.8
Obstetrics and gynaecology	1441	95	2.4	6.6
Surgery	1413	90	2.3	6.4
Palliative medicine	57	3	0.1	5.3
Urology	391	16	0.4	4.1
Neurosurgery	133	5	0.1	3.8
Others[c]	556	20	0.5	3.6
Orthopaedics	1399	46	1.2	3.3
Pain medicine	154	4	0.1	2.6
Plastic surgery	161	4	0.1	2.5
Anaesthesiology	1794	43	1.1	2.4
Vascular surgery	156	3	0.1	1.9
Hand surgery	126	2	0.1	1.6
Child and adolescent surgery	74	1	0.0	1.4
Radiology	1503	10	0.3	0.7
Laboratory specialities	912	3	0.1	0.3
Gynaecological oncology	52	0	0.0	0.0
Thoracic surgery	129	0	0.0	0.0

[a] Internal medicine also includes endocrinology, gastro-enterology and nephrology.

[b] Otolaryngology also includes audiology and phoniatrics.

[c] Others include clinical pharmacology, occupational- and environmental medicine, and school health care medicine

S: Table 2B Adverse drug reaction reporting rates (number of ADR reports per 100 specialists) among different specialties year 2023

**Specialty**	**Number of specialists 2023**	**Number of ADR reports 2023**	**% of all ADR reports 2023**	**Reporting rate 2023 (Number of ADR reports per 100 specialists)**
Rheumatology	301	120	3.3	39.9
Neurology	553	189	5.3	34.2
Internal medicine[a]	2280	723	20.1	31.7
Otolaryngology[b]	751	217	6.0	28.9
Dermatology	497	121	3.4	24.3
Infection medicine	563	131	3.6	23.3
Haematology	312	55	1.5	17.6
Emergency medicine	470	82	2.3	17.4
Pulmonary medicine	354	59	1.6	16.7
GPs	6976	1038	28.8	14.9
All Specialties	32394	3598	100.0	11.1
Oncology	577	64	1.8	11.1
Psychiatry	2160	197	5.5	9.1
Cardiology	1121	98	2.7	8.7
Paediatrics	1818	142	3.9	7.8
Ophthalmology	851	60	1.7	7.1
Child and adolescent psychiatry	466	31	0.9	6.7
Obstetrics and gynaecology	1512	80	2.2	5.3
Geriatrics	563	26	0.7	4.6
Surgery	1502	63	1.8	4.2
Rehabilitation medicine	159	6	0.2	3.8
Others[c]	479	18	0.5	3.8
Pain medicine	154	5	0.1	3.2
Urology	476	9	0.3	1.9
Gynaecological oncology	55	1	0.0	1.8
Orthopaedics	1568	22	0.6	1.4
Anaesthesiology	1997	23	0.6	1.2
Thoracic surgery	118	1	0.0	0.8
Radiology	1656	11	0.3	0.7
Palliative medicine	219	1	0.0	0.5
Laboratory specialities	1140	5	0.1	0.4
Child and adolescent surgery	83	0	0.0	0.0
Hand surgery	142	0	0.0	0.0
Vascular surgery	194	0	0.0	0.0
Neurosurgery	135	0	0.0	0.0
Plastic surgery	172	0	0.0	0.0

[a] Internal medicine also includes endocrinology, gastro-enterology and nephrology.

[b] Otolaryngology also includes audiology and phoniatrics.

[c] Others include clinical pharmacology, occupational- and environmental medicine, and school health care medicine
